# The myth of psychological debriefings during the corona pandemic

**DOI:** 10.7189/jogh.10.020344

**Published:** 2020-12

**Authors:** Roel Van Overmeire

**Affiliations:** Mental Health and Wellbeing research group, Vrije Universiteit Brussel, Belgium

During the corona pandemic, health care personnel can encounter stressful events such as: an increased exposure to deceased people, an overwhelming workload, and no time to recover [1,2]. After such events, psychological debriefing moments are often organized and recommended in some scientific literature (eg, Walton, Murray, and Christian [1]; Khan, et al [2]). Even more so, psychological debriefings are found to be so common sense, that authors sometimes leave out references that provide the supportive background literature regarding the effectiveness of such debriefings (eg, Walton, Murray, & Christian [1]). Which is not surprising, as there is hardly any evidence for the effectiveness of psychological debriefings [3-5].

This knowledge is concerning for two reasons. First, it indicates the presence of a bottle neck in the information flow to the involved population. A finding that can be supported by a critique last year (see Burchill’s letter to the editor [6]) citing the same observation, albeit in another form. Second, health care providers today operate in an unprecedented crisis leading to an increasing workload. Therefore, it is worrying that caregivers lack appropriate recommendations for support during crisis events.

A psychological debriefing, with the most popular and common being ‘critical incident stress debriefing’, has the goal to process traumatic and stressful events in health care providers, so that mental health consequences can be avoided. In group, participants are guided by session leaders and psycho-educational information is provided in order to normalize people’s reaction to certain events. Group sessions are the most common form of psychological debriefings and take place normally 48 to 72 hours after a traumatic event [7].

However, while psychological debriefings are welcomed by people participating in them [8], it is not a direct confirmation of its effectiveness [9]. While one meta-analysis found a positive effect [10], most other large-scale studies state that there is no evidence for the usefulness of such debriefings. One systematic review even recommends terminating the application of these debriefings, as there are indications that they are harmful for the mental health of people participating in them [3].

Furthermore, most studies on debriefings are outdated, the majority being around 20 years old. However, there are two recent reviews on debriefings, which are both cited by Khan, et al [2] to advocate the further integration of these debriefing principles. Interestingly, both reviews actually contradict Khan, et al [2]. The systematic review by Roberts, et al [4] stated that there is no clear evidence for the usefulness of psychological debriefings. Also, the scoping review by Harder, Lemoine, Harwood [5] clearly stated a lack of evidence available to recommend debriefings in the clinical practice. Which essentially undermines the point Khan, et al. [2] wish to prove in favour of debriefings.

In conclusion, there is an insufficient amount of recent research available to demonstrate the added value of psychological debriefings against the development of mental health problems. Nevertheless, they are still recommended with sometimes no citation for their usefulness, nor citations of articles providing contradictory conclusions. Although, it is important to provide health care workers with a solid supportive framework, time should be invested in searching proven interventions rather than psychological debriefings, which remain highly questionable. Continuing on the latter, it is surprising that such debriefings are still recommended, as there are numerous indications that psychological debriefings lack beneficial effects in supporting mental health providers during extremely stressful events and should instead be avoided [3].

**Figure:**
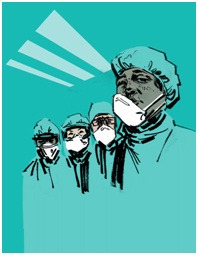
Photo: Source: Kevin Kobsic; Global Call Out To Creatives – help stop the spread of COVID-19 (https://unsplash.com/photos/N1caHdFQ734).

It is understandable that the instinct to provide help predominates in such pressing times. But as researchers, there should still be the need to look into the scientific background surrounding these interventions. Thereby, it is important that the information is based on accuracy and scientific literature, rather than unscientific recommendations, as would seem to be the case in recommending psychological debriefings. Therefore, it seems timelier to shift the focus from recommending psychological debriefings to seeking evidence-based interventions to help prevent mental health implications on health care providers. There are numerous such evidence-based interventions. For example, systematic reviews have shown that interventions focussing on both mental and physical health or interventions for particular anxieties have research evidence [11]. Another example are interventions that are based on Mindfulness-Based Stress Reduction, with nurses showing lower depression, stress, burnout and anxiety rates as a result [12]. Finally, health care personnel can be trained to increase their resilience during crisis moments, by training them in mindfulness meditation, by which they are able to decrease stress levels without interventions [13].
